# Relation of mitral valve morphology and motion to mitral regurgitation severity in patients with mitral valve prolapse

**DOI:** 10.1186/1476-7120-10-3

**Published:** 2012-01-27

**Authors:** Mario Sénéchal, Nicolas Michaud, Jimmy MacHaalany, Mathieu Bernier, Michelle Dubois, Julien Magne, Christian Couture, Patrick Mathieu, Olivier F Bertrand, Pierre Voisine

**Affiliations:** 1 Department of Cardiology, Institut Universitaire de Cardiologie et de Pneumologie de Québec, Laval University, Chemin Sainte-Foy, Quebec, G1V 4G5, Canada; 2 Department of Anatomo-pathology, Institut Universitaire de Cardiologie et de Pneumologie de Québec, Laval University, Chemin Sainte-Foy, Quebec, G1V 4G5, Canada; 3 Department of Cardiovascular Surgery, Institut Universitaire de Cardiologie et de Pneumologie de Québec, Laval University, Chemin Sainte-Foy, Quebec, G1V 4G5, Canada

**Keywords:** mitral regurgitation, mitral valve, echocardiography, mitral valve prolapse

## Abstract

**Background:**

Mitral valve thickness is used as a criterion to distinguish the classical from the non-classical form of mitral valve prolapse (MVP). Classical form of MVP has been associated with higher risk of mitral regurgitation (MR) and concomitant complications. We sought to determine the relation of mitral valve morphology and motion to mitral regurgitation severity in patients with MVP.

**Methods:**

We prospectively analyzed transthoracic echocardiograms of 38 consecutive patients with MVP and various degrees of MR. In the parasternal long-axis view, leaflets length, diastolic leaflet thickness, prolapsing depth, billowing area and non-coaptation distance between both leaflets were measured.

**Results:**

Twenty patients (53%) and 18 patients (47%) were identified as having moderate to severe and mild MR respectively (ERO = 45 ± 27 mm^2 ^vs. 5 ± 7 mm^2^, p < 0.001). Diastolic leaflet thickness was similar in both groups (5.5 ± 0.9 mm vs. 5.3 ± 1 mm, p = 0.57). On multivariate analysis, the non-coaptation distance (OR 7.9 per 1 mm increase; 95% CI 1.72-37.2) was associated with significant MR. Thick mitral valve leaflet as traditionally reported (≥ 5 mm) was not associated with significant MR (OR 0.9; 95% CI 0.2-3.4).

**Conclusions:**

In patients with MVP, thick mitral leaflet is not associated with significant MR. Leaflet thickness is probably not as important in risk stratification as previously reported in patients with MVP. Other anatomical and geometrical features of the mitral valve apparatus area appear to be much more closely related to MR severity.

## Background

Mitral valve thickness ≥ 5 mm is used as a criterion to distinguish classical from non-classical form of mitral valve prolapse (MVP). Classical form of MVP has been associated with a higher risk of mitral regurgitation (MR) and cardiovascular complications [1-8]. However, increased leaflet thickness is frequently observed in MVP even without MR [4], and thus might be an imperfect criterion to stratify the clinical risk of patients with MVP. Previous studies on MVP and mitral valve morphology have not used quantitative methods to assess MR severity. Most of them used semi-quantitative evaluations of MR such as jet-to-left-atrial area ratios. Moreover, the relation between MR and the billowing area or the non-coaptation distance between leaflets has not been evaluated. We sought to determine the relation of mitral valve morphology and motion to mitral regurgitation severity in patients with MVP.

## Methods

Between January 2010 and September 2010 at the Institut Universitaire de Cardiologie et de Pneumologie de Québec, we prospectively analyzed transthoracic echocardiography of consecutive patients with known or suspected mild to severe MR (asymptomatic or symptomatic). Only patients with isolated posterior MVP were included (Additional file 1). Leaflet displacement ≥ 2 mm across the annulus plane in the parasternal long-axis view was mandatory for the diagnosis of MVP. Patients in whom MR might be explained by mechanisms other than prolapse were excluded: left ventricular (LV) ejection fraction < 35% or end-diastolic LV diameter > 65 mm (functional MR), previous myocardial infarction (ischemic MR), rheumatic mitral valve disease or endocarditis (organic MR). Patients with flail leaflet, prior mitral annuloplasty or poor echogenicity were also excluded. The final study group was composed of 38 patients. All patients provided informed consent. The study protocol conforms to the ethical guidelines of the 1975 Declaration of Helsinki and was approved by local ethics committee

**Figure 1:**
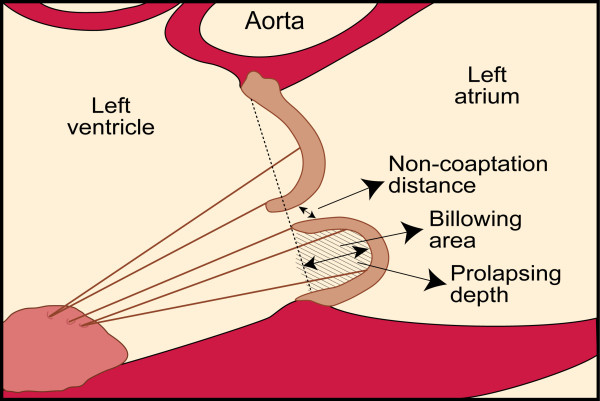
**Diagram showing measurements of mitral valve geometry of a posterior leaflet prolapse from parasternal long-axis view**.

Complete two-dimensional and Doppler transthoracic echocardiography examinations with commercially available echocardiographic systems (Sonos 5500, 7500 or iE33, Philips Medical Systems, Amsterdam, the Netherlands) were performed by experienced sonographers. All measurements were performed off line with Xcelera Echo Lab Management (Philips Medical Systems, Amsterdam, the Netherlands). Diastolic leaflet thickness, leaflets length, prolapsing depth, billowing area and non-coaptation distance between both leaflets were measured in the parasternal long-axis view [4]. Leaflet length was measured from the tip of the leaflets to the insertion at the annulus in diastole. Mitral leaflet thickness in diastole was measured from the leading to the trailing edge of the thickest area at the mid portion of the leaflet, excluding focal areas of thickness [7-11]. Prolapsing depth was defined as the distance between the annular plane and the maximally prolapsing leaflet. Billowing area was measured as the area between the annular plane and the leaflet, at its maximal excursion. The non-coaptation distance was the maximal distance between both leaflet edges, usually at the end of the systole (Figure 1, Additional file 2) [4]. The LV end-diastolic and end-systolic diameters were measured using M-mode in the parasternal long-axis view. LV end-diastolic and end-systolic volumes and LV ejection fraction were determined by the modified biplane Simpson method [12]. MR was detected from color Doppler echocardiography in the apical four-chamber view. MR severity was assessed quantitatively by ERO measurement as previously described [13].

Two-dimensional echocardiograms from 20 patients with MVP were analyzed independently by two investigators (MS, NM). The coefficient of variability was calculated by dividing the standard deviation of the mean difference by the mean value of the specific parameter (leaflet thickness 9%, non coaptation distance 5%, prolapsing depth 7%, and billowing area 9%). Absolute values correlated closely between the 2 investigators with *r *values ranging from 0.82 to 0.98. Patients were separated into 2 groups depending on the presence of mild MR (ERO < 20 mm^2^) or moderate to severe MR (ERO ≥ 20 mm^2^). The numeric mean and standard deviation of the diastolic leaflet thickness, leaflet length, prolapsing depth, billowing area, non-coaptation distance between leaflets and ERO were calculated for both groups. Student T test was performed to identify significant differences between groups. Differences between proportions were evaluated by the chi-square test. A p value < 0.05 was considered statistically significant. Univariate and multivariate logistic regression analyses were performed using the presence of significant MR as the dependent variable. Correlation between continuous variables was assessed with Pearson's Correlation Coefficient.

## Results

**Figure 2:**
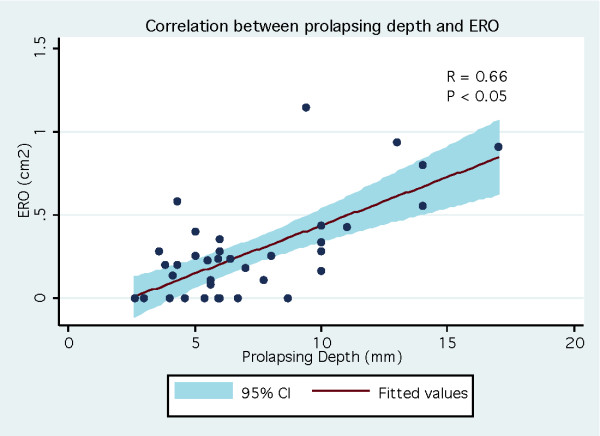
**Correlation between prolapsing depth and ERO (effective regurgirtant orifice)**.

**Figure 3:**
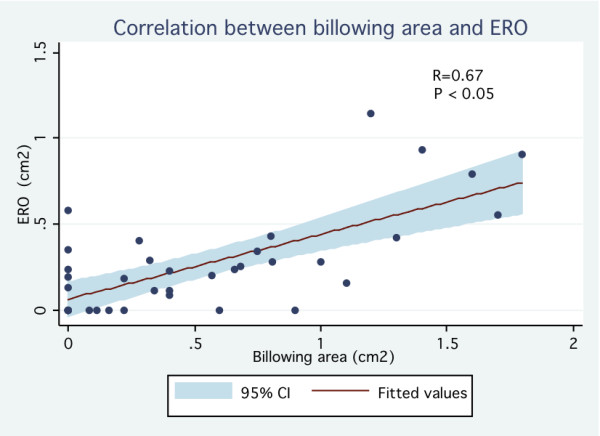
**Correlation between billowing area and ERO (effective regurgitant orifice)**.

**Figure 4:**
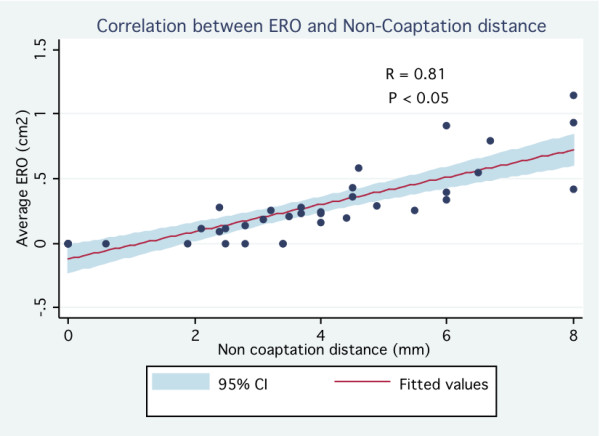
**Correlation between non-coaptation distance and ERO (effective regurgitant orifice)**.

**Figure 5:**
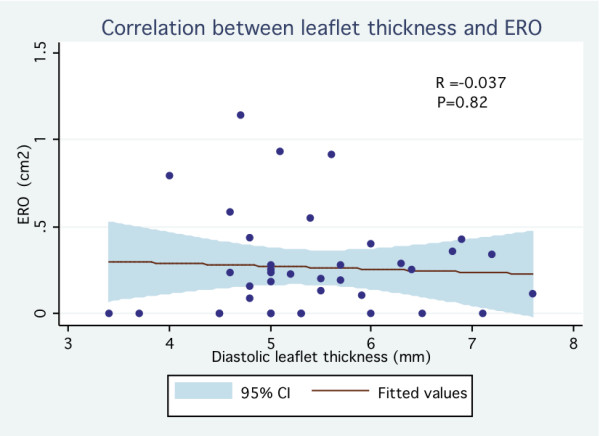
**Correlation between leaflet thickness and ERO (effective regurgitant orifice)**.

Of the 38 patients, 20 patients (53%) and 18 patients (47%) were identified as having mild MR and moderate to severe MR respectively (ERO = 45 ± 27 mm^2 ^vs. 5 ± 7 mm^2^, p < 0.001) (Table 1). Patients with moderate to severe MR were older (61 ± 14 years vs. 41 ± 18 years, p < 0.001) and had a slightly larger LV end diastolic diameter (49 ± 6 mm vs. 45 ± 4 mm, p = 0.03). There was no significant difference between both groups regarding LV end systolic diameter and LV ejection fraction. With regard to mitral valve morphology, patients with ERO ≥ 20 mm^2 ^had a longer posterior leaflet (18.4 ± 4.2 mm vs. 13.6 ± 3.6 mm, p < 0.001) and greater prolapsing depth (8.4 ± 3.8 mm vs. 5.5 ± 2.0 mm, p = 0.007). Diastolic leaflet thickness was similar between both groups (5.5 ± 0.9 mm vs. 5.3 ± 1.0 mm, p = 0.57). Billowing area tended to be different in patients with moderate to severe MR (1.0 ± 0.6 cm^2 ^vs. 0.7 ± 0.4 cm^2^, p = 0.08). As expected, the non-coaptation distance between leaflets was greater among those with ERO ≥ 20 mm^2 ^(5.0 ± 1.6 mm vs. 2.0 ± 2.5 mm, p < 0.001). Proportion patients with classical form of MVP (thickened leaflets ≥ 5 mm) was similar in both groups (30% vs. 33%, p = 0.82). On univariate analysis, the strongest predictors of significant MR were the non-coaptation distance (OR 6.2 per 1 mm increase; 95% CI 1.72-22.3) and the prolapsing depth (OR 1, 4 per 1 mm increase; 95% CI 1.1-1.7) Thick mitral valve leaflet as traditionally reported (≥ 5 mm) was not associated with significant MR (OR 0.9; 95% CI 0.2-3.4) (Table 2). On multivariate analysis, the only feature statistically associated with significant MR was the non-coaptation distance (OR 7.9 per 1 mm increase; 95% CI 1.7-37.2). In the whole MVP population, ERO correlated well with prolapsing depth (r = 0.66, p < 0.05) (Figure 2), billowing area (r = 0.67, p < 0.05) (Figure 3) and particularly with the non-coaptation distance between leaflets (r = 0.81, p < 0.05) (Figure 4). No significant correlation was demonstrated between ERO and leaflet thickness (r = -0.037, p = 0.82) (Figure 5). In the subgroup of patients with ERO ≥ 20 mm^2 ^(n = 20), 8 (40%) patients had mitral valve surgery because of symptomatic MR; 2 patients had mitral valve repair and 6 had mitral valve replacement. In those patients (n = 8), non-coaptation distance (6.3 mm vs. 4.5 mm, p = 0.01), posterior leaflet length (20.4 mm vs. 17.2 mm, p = 0.01) and prolapsing depth (10.6 mm vs. 7.0 mm, p = 0.03) were statistically different from those of patients who did not require surgery despite having an ERO ≥ 20 mm^2^. However, leaflet thickness was not statistically different between the 2 groups (5.5 mm vs. 5.5 mm, p = 0.99).

**Table 1:** Patients characteristics according to ERO

	ERO < 20 mm n = 18	ERO ≥ 20 mm n = 20	P Value
**Age (y)**	41 ± 18	61 ± 14	< 0.001
**Gender (male)**	8/18 (44%)	12/20 (60%)	0.33
**Systolic blood pressure (mmHg)**	126 ± 19	133 ± 20	0.39
**Diastolic blood pressure (mmHg)**	79 ± 8	78 ± 9	0.9
**Left ventricular end diastolic diameter (mm)**	45 ± 4	49 ± 6	0.03
**Left ventricular end systolic diameter (mm)**	28 ± 5	29 ± 6	0.65
**Posterior leaflet length (mm)**	13.6 ± 3.6	18.4 ± 4.2	< 0.001
**Left ventricular ejection fraction (%)**	58 ± 7	60 ± 6	0.3
**Diastolic thickness (mm)**	5.3 ± 1.0	5.5 ± 0.9	0.57
**Non Coaptation distance (mm)**	2.0 ± 2.5	5.0 ± 1.6	< 0.001
**Billowing area (cm^2^)**	0.7 ± 0.4	1.0 ± 0.6	0.08
**Prolapsing depth (mm)**	5.5 ± 2.0	8.4 ± 3.8	0.007
**ERO (mm^2^)**	5 ± 7	45 ± 27	< 0.001

ERO: effective regurgitant orifice

**Table 2:** Mitral valve features associated with significant regurgitation (ERO ≥ 20 mm^2^)

	Univariate analysis		Multivariate Analysis	
	OR	P Value	OR	P Value
**Mitral leaflet thickness **≥ **5 mm**	0.9	0.69	1.7	0.7
**Prolapsing depth per 1 mm increase**	1.4	0.02	3.3	0.13
**Billowing area per 1 mm^2 ^increase**	3.7	0.09	1.0	0.10
**Non-coaptation distance per 1 mm increase**	6.2	0.006	7.9	0.009

## Discussion

Results from this study contradict the assumption that mitral leaflet thickness is a significant criterion for the characterization of the classical form of MVP and, by extension, the alleged relation with the severity of MR and its consequent cardiovascular complications. In fact, the present study suggests that leaflet thickness is similar in subjects with MVP, without regard to MR severity. On the other hand, other features of mitral valve anatomy and geometry such as the length of the posterior leaflet, the depth of leaflet prolapse and, as expected by direct impact on ERO, the non-coaptation distance between the leaflets, were all found to be significantly correlated with MR severity. Thus the present study provides additional data linking the presence of abnormal mitral geometry with MR, a complication of MVP known to be associated with an increased risk of adverse clinical events. This the first study on MVP where combined anatomical (i.e. leaflet thickness and length) and geometrical mitral valve assessment (i.e. non-coaptation distance, prolapsing depth, billowing area) were done in addition with quantification of MR severity with the use of ERO measurement.

The present study both partly confirms but also substantially diverges from conclusions from previous reports [2,4-8,14-16]. Consistent with our findings, Weissman *et al. *[4] have already reported that the usefulness of leaflet thickness as a marker of MR severity is limited by its high prevalence in patients with clinically mild MR. In that study, leaflet thickness of different zones were supranormal in more than 50% of MVP patients with or without severe MR. More recently, Freed *et al. *[15] demonstrated that MR severity was similar when comparing patients with classical MVP (thickness ≥ 5 mm) and patients with non-classical MVP. In a prospective study including 285 patients during a follow up ≥ 4 years, Avierinos *et al. *[2] showed that the progression of MR was observed in all subsets, irrespective of age, gender, prolapsed localization, leaflet thickness, and initial mitral MR grade. Moreover, that study showed that patients with moderate MR were more likely to progress to severe MR, and that MR progression results in an excess long-term complication rate, independent of confounding variables. Pini *et al. *[5] have shown that patients with MVP complicated by significant MR are more likely to have billowing and leaflet elongation than are MVP patients without MR. Although all features of mitral valve geometry alteration were not measured and excessive leaflet thickness was not specifically addressed in that study, those findings and those of our study are concordant. Accordingly, other studies have distinguished mitral valve billowing, in which leaflet apposition is normal, from MVP in which the leaflets fail to appose properly so that MR occurs [4,6,16]. Grayburn et *al*. [16] demonstrated that abnormal mitral leaflet coaptation on 2 dimensional echocardiography was strongly associated with the presence of MR, with a prevalence of 71% (15 of 21 patients) and 20% (5 of 25 patients, p < 0.05) in patients with and without significant MR, respectively.

Contrastingly, Malkowski *et al. *[6] concluded that leaflet thickness assessment is fundamental to the definition and stratification of patients with MVP. This conclusion was based on the demonstration of increased anterior leaflet thickness in patients with MVP and significant MR in comparison to patients without significant MR (5.8 ± 0.8 mm vs. 5.2 ± 0.7 mm, p < 0.05). Despite statistical significance, a 0.6 mm average difference in leaflet thickness can hardly be considered clinically relevant. Furthermore, interobserver variability for the measurement of leaflet was 13%, suggesting that the measurement of leaflet thickness in patients with MVP could not be used in a reproducible and useful manner in clinical practice. In another study Marks *et al. *[8] showed that in a selected population of patients with MVP, those with the classical form were more frequently diagnosed with moderate to severe MR than patients without the classical form. Importantly, MR severity was quantified only semi-quantitatively by measurement of the regurgitant jet within the atrium in that population. Moreover, the definition of the classical form of MVP used in that study not only included the usual criterion of thickness (≥ 5 mm) but also leaflet redundancy, which was qualitatively defined as a disproportionate increase in the circumference of the leaflet relative to chamber size, so that they had an undulant appearance during valvular opening. We suggest that this is basically a relatively simpler qualitative description of the alterations in mitral valve geometry that we have measured quantitatively in our study. By combining both leaflet length and thickness measurement and quantitative assessment of valve redundancy during MR (i.e. alteration of the mitral valve geometry), we were able to evaluate the respective importance and impact of these anatomical and geometrical criteria on the severity of MR in a population of patients with MVP.

## Limitation section

Conclusions from our data are limited by the relatively small size of the patient cohort. Despite the apparent absence of link between leaflet thickness and the importance of MR, the possible association with an increased risk of other complications such as endocarditis was not analyzed in our study and can therefore not be excluded. However it should be mentioned that in a previous study where a higher risk of endocarditis was linked to the classical form of MVP, mitral valve redundancy was included with mitral leaflet thickness in the definition of classical MVP [8]. It remains uncertain whether the prevalence of infective endocarditis in patients with MVP is mainly influenced by mitral valve thickness and/or by mitral leaflet redundancy.

## Conclusions

In patients with MVP, thickness of mitral leaflets is not associated with the severity of MR. Leaflet thickness is probably not as important in risk stratification as previously reported in patients with MVP. Other anatomical and geometrical features of the mitral apparatus such as the length of the posterior leaflet, the depth of leaflet prolapse, the non-coaptation distance between the leaflets and the billowing area appear to be much more closely related to the severity of MR and should be given more important consideration for risk stratification in these patients.

## Competing interests

The authors declare that they have no competing interests.

## Authors' contributions

MS is the principal investigator, he concept the study, collected, analysed and interpreted the data and drafted the article, NM, OFB and JM collected and interpreted the data and participated in drafting the manuscript, MB did the statistical analysis and revised the manuscript, MD collected the data and revised the manuscript, JM collected data, CC did a critical revision of the article, PM did mitral valve replacement, PV did mitral replacement, collected data and participated in drafting the manuscript. All authors approved the article.

## Supplementary Material

Additional file 1 **Transthoracic echocardiography of a patient with mitral valve prolapse**. Transthoracic echocardiography of a patient with mild MR (ERO = 11 mm^2^) and leaflet thickness > 5 mm. Click here for file

Additional file 2 **Non-coaptation distance measurement of a patient with mitral valve prolapse**. Measurement of a non-coaptation distance (2, 4 mm) in the same patient with mild MR. Click here for file
