# Supplementary figures and images for: Relation of mitral valve morphology and motion to mitral regurgitation severity in patients with mitral valve prolapse
Source: Cardiovasc Ultrasound. 2012 Jan 27;10:3. doi: 10.1186/1476-7120-10-3 (PMC3296553; doi:10.1186/1476-7120-10-3)

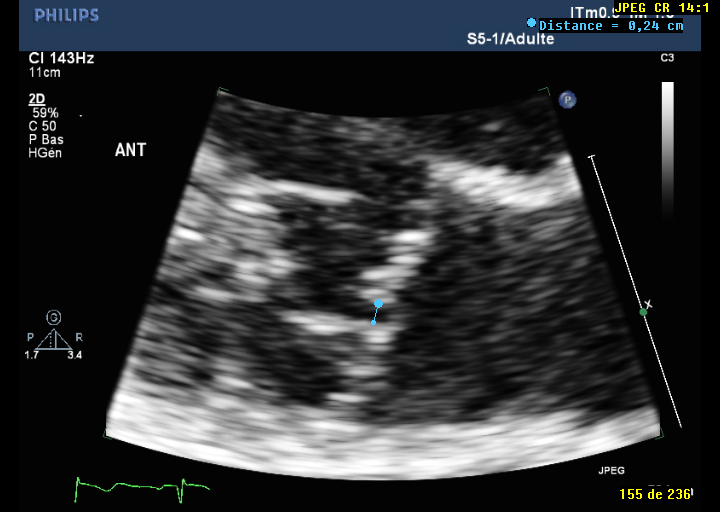

Supplement: Additional file 2 — Non-coaptation distance measurement of a patient with mitral valve prolapse. Measurement of a non-coaptation distance (2, 4 mm) in the same patient with mild MR. [file 1476-7120-10-3-S2.BMP]
